# Mechanisms of antimicrobial resistance in Gram-negative bacilli

**DOI:** 10.1186/s13613-015-0061-0

**Published:** 2015-08-12

**Authors:** Étienne Ruppé, Paul-Louis Woerther, François Barbier

**Affiliations:** Department of Infectious Diseases, Genomic Research Laboratory, Geneva University Hospitals, Geneva, Switzerland; Department of Microbiology, Gustave-Roussy Institute, Villejuif, France; Medical Intensive Care Unit, La Source Hospital - CHR Orléans, Orléans, France

**Keywords:** Enterobacteriaceae, *Pseudomonas aeruginosa*, *Acinetobacter baumannii*, *Stenotrophomonas maltophilia*, Antimicrobial resistance, Extended-spectrum beta-lactamase, Carbapenemase, Colistin, Intestinal microbiota, Intensive care unit

## Abstract

The burden of multidrug resistance in Gram-negative bacilli (GNB) now represents a daily issue for the management of antimicrobial therapy in intensive care unit (ICU) patients. In *Enterobacteriaceae*, the dramatic increase in the rates of resistance to third-generation cephalosporins mainly results from the spread of plasmid-borne extended-spectrum beta-lactamase (ESBL), especially those belonging to the CTX-M family. The efficacy of beta-lactam/beta-lactamase inhibitor associations for severe infections due to ESBL-producing *Enterobacteriaceae* has not been adequately evaluated in critically ill patients, and carbapenems still stands as the first-line choice in this situation. However, carbapenemase-producing strains have emerged worldwide over the past decade. VIM- and NDM-type metallo-beta-lactamases, OXA-48 and KPC appear as the most successful enzymes and may threaten the efficacy of carbapenems in the near future. ESBL- and carbapenemase-encoding plasmids frequently bear resistance determinants for other antimicrobial classes, including aminoglycosides (aminoglycoside-modifying enzymes or 16S rRNA methylases) and fluoroquinolones (Qnr, AAC(6′)-Ib-cr or efflux pumps), a key feature that fosters the spread of multidrug resistance in *Enterobacteriaceae*. In non-fermenting GNB such as *Pseudomonas aeruginosa*, *Acinetobacter baumannii* and *Stenotrophomonas maltophilia*, multidrug resistance may emerge following the sole occurrence of sequential chromosomal mutations, which may lead to the overproduction of intrinsic beta-lactamases, hyper-expression of efflux pumps, target modifications and permeability alterations. *P. aeruginosa* and *A. baumannii* also have the ability to acquire mobile genetic elements encoding resistance determinants, including carbapenemases. Available options for the treatment of ICU-acquired infections due to carbapenem-resistant GNB are currently scarce, and recent reports emphasizing the spread of colistin resistance in environments with high volume of polymyxins use elicit major concern.

**Electronic supplementary material:**

The online version of this article (doi:10.1186/s13613-015-0061-0) contains supplementary material, which is available to authorized users.

## Background

The burden of antimicrobial resistance in Gram-negative bacilli (GNB) is a daily challenge to face for intensive care unit (ICU) physicians. Indeed, GNB are responsible for 45–70% of ventilator-associated pneumonia (VAP) [1], 20–30% of catheter-related bloodstream infections [2], and commonly cause other ICU-acquired sepsis such as surgical site or urinary tract infections (UTI) [3]. In such situations, the timely administration of adequate antibiotic coverage is a crucial determinant of patient outcome, especially when criteria for severe sepsis are present [4]. Nevertheless, alarming resistance rates are now reported worldwide, and rising trends may elicit concerns for the coming years [2, 3, 5–9]. Almost exclusively restricted to the hospital setting till the beginning of the century, this issue increasingly applies for patients with healthcare-associated [10, 11] and even community-acquired infections [12–14]. *Enterobacteriaceae* and non-fermenting GNB (*Pseudomonas aeruginosa*, *Acinetobacter baumannii* and *Stenotrophomonas maltophilia*) account for the major part of the problem [15].

Antimicrobial resistance in GNB results from the expression of antibiotic-inactivating enzymes and non-enzymatic mechanisms [16]. Both may be intrinsically expressed by a given species (chromosomal genes), or acquired by a subset of strains as a consequence of two distinct albeit not mutually exclusive genetic events:

1. Mutations in chromosomal genes resulting in an increase in the expression of intrinsic resistance mechanisms (either antibiotic-inactivating enzymes or efflux pumps), permeability alterations by loss of outer membrane porins, or target modifications;
2. Horizontal transfers of mobile genetic elements (MGEs) carrying resistance genes, most notably plasmid-encoding beta-lactamases, aminoglycosides-modifying enzymes (AMEs), or non-enzymatic mechanisms such as Qnr for fluoroquinolone resistance in *Enterobacteriaceae*. Since these plasmids commonly bear multiple resistance determinants, a single plasmid conjugation may suffice to confer a multidrug resistance phenotype to the recipient strain.

The mechanisms of antimicrobial resistance in GNB may interfere with several facets of antibiotic stewardship algorithms in critically ill patients, including the choice of empirical regimen, available options for de-escalation, and the management of clinical failure due to the emergence of resistance under therapy [17, 18]. In this concise review, we sought to summarize the current knowledge on resistance mechanisms and epidemiologic trends in the main clinically relevant species belonging to *Enterobacteriaceae* and non-fermenting GNB, and make the connection with the use of antimicrobial therapy in the ICU.

## Review

### Current trends in the global epidemiology of multidrug-resistant GNB

Each given ICU has its own bacterial ecology, which may fluctuate owing to antibiotic use policies, patient recruitment and sporadic outbreaks. Yet, data from large surveillance networks yield a general overview of resistance rates in GNB causing ICU-acquired infections (Table 1). Following a decade of steady rise [19], rates of resistance to third-generation cephalosporins (3GC) in *Enterobacteriaceae* are now constantly above 10% and may reach 70% in certain settings [3, 5, 9]. This situation mainly results from the rapid spread of extended-spectrum beta-lactamase (ESBL)-producing strains, which currently account for 15–25% of *Enterobacteriaceae* isolated from clinical samples in critically ill patients [2, 9]. Far more worrying is the on-going dissemination of carbapenem-resistant *Enterobacteriaceae* (CRE), with an overall prevalence of 2–7% in ICUs in Europe, Asia and the United States [3, 5, 8, 9]. This issue appears especially critical for *Klebsiella pneumoniae*, with carbapenem resistance rates above 25% in several Southern European countries such as Italy or Greece [5]. Current rates of ceftazidime and carbapenem resistance in *P. aeruginosa* range from 20 to 40%. Multidrug resistance (i.e., resistance to at least three antimicrobial classes out of piperacillin–tazobactam, ceftazidime, fluoroquinolones, aminoglycosides and carbapenems) and extensive drug resistance (i.e., resistance to the five classes mentioned above) accounted for, respectively, 13 and 4% of *P. aeruginosa* isolates reported to the European Center for Disease Prevention and Control in 2013 [5]. Resistance rates are equally on the rise in *A. baumannii*, with 40 to 70% of isolates responsible for ICU-acquired infections being carbapenem resistant [2, 3, 8, 9].

**Table 1:** Rates of antimicrobial resistance in Gram-negative bacilli responsible for hospital-acquired infections

Study/surveillance network	INICC [3]	SENTRY [9]	ANSRPRG [8]	EARS-NET [5]
Geographic area	International (36 countries)	International (Europe/USA)	International (Asia)	International (Europe)
Study years	2004–2009	2009–2011	2008–2009	2013
Setting	ICU	ICU	ICU/non-ICU	ICU/non-ICU
Type of hospital-acquired infections	Catheter-related infections and ventilator-associated pneumonia	All (pooled)	Pneumonia	Bloodstream infections
Species/antimicrobial				
*Escherichia coli*				
Fluoroquinolones	53%	30%	–	11–52%
3GC	67%	13%	–	5–40%
Carbapenems	4%	<1%	–	0–3%
*Klebsiella pneumoniae*				
Fluoroquinolones	–	17%	31%	0–70%
3GC	72%	19%	43%	0–70%
Carbapenems	7%	4%	2%	0–59%
*Pseudomonas aeruginosa*				
Fluoroquinolones	45%	30%	30%	0–53%
Aminoglycosides	28%	17%^a^	–	0–51%
Piperacillin–tazobactam	39%	32%	37%	0–55%
Ceftazidime	–	27%	35%	0–44%
Carbapenems	45%	30%^b^	30%	3–60%
*Acinetobacter baumannii*				
Ceftazidime	–	63%	–	–
Carbapenems	63%	57%^b^	67%	0–90%

*ICU* intensive care unit, *3GC* third-generation cephalosporins.

^a^Indicator: gentamicin.

^b^Indicator: meropenem.

### Antimicrobial resistance in *Enterobacteriaceae*

#### Resistance to beta-lactams

**Fig. 1:**
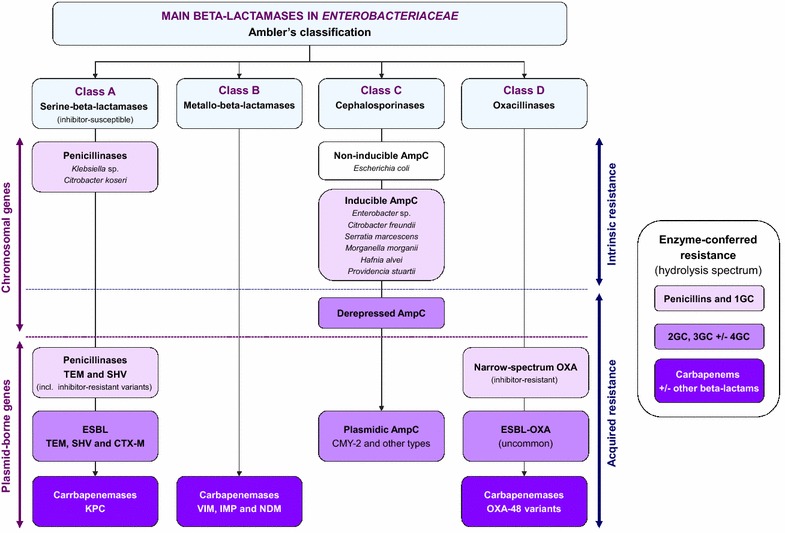
Intrinsic and acquired beta-lactamases in *Enterobacteriaceae*.

Beta-lactamase production is the main mechanism of beta-lactam resistance in *Enterobacteriaceae* (Fig. 1) [20]. These highly diversified enzymes hydrolyze beta-lactams in the periplasmic space, thus preventing penicillin-binding protein inhibition. *Enterobacteriaceae* are usually classified with regard to their intrinsic beta-lactamase content (Additional file 1: Table S1). A peculiar phenotype is observed in species that produce an inducible, chromosome-encoded AmpC cephalosporinase, notably *Enterobacter* sp., *Citrobacter freundii*, *Hafnia alvei*, *Morganella morganii*, *Serratia marcescens* and *Providencia* sp. [21]. AmpC is strongly induced by amoxicillin, clavulanic acid, cefoxitin and first-generation cephalosporins (1GC), which results in intrinsic resistance. Carbapenems are also potent inducers but remain active due to lack of significant AmpC-mediated hydrolysis, whereas other beta-lactams are weaker inducers. Infections caused by wild-type-inducible AmpC producers should be preferably treated by ticarcillin or piperacillin: 3GC, while active, must be avoided owing to a higher risk for selecting resistant mutants (see below) and a larger ecological impact [22–24]. Mutations in the induction system may permanently lead to AmpC overexpression (i.e., derepression). Of note, these mutations happen spontaneously and are only selected—but not prompted—by beta-lactams [25]. AmpC-hyperproducing mutants are resistant to penicillins, aztreonam, 3GC and even ertapenem when the enzyme is massively expressed [26] : imipenem and meropenem remain the most active beta-lactams, although cefepime stands as a valuable carbapenem-sparing option when tested susceptible and provided that the source of infection is controlled [27, 28]. The selection of a resistant mutant must be ruled out when a VAP or another infection due to wild-type-inducible AmpC producers does not improve, or relapse, under a first-line beta-lactam: in this case, a new sample should be obtained for susceptibility control [29]. Overall, AmpC hyperproducers account for 50–65% of 3GC-resistant *Enterobacteriaceae* recovered from carriage or clinical specimen in ICU with high prevalence of ESBL-producing strains [2, 30, 31]. Besides, the genome of *Escherichia coli* holds a very low-level expressed chromosomal AmpC not regulated by the induction system mentioned above [21]. Hyperproducing mutants may occasionally emerge; however, this mechanism of 3GC resistance remains anecdotal in *E. coli* when compared to ESBL [32].

The dissemination of plasmid-borne beta-lactamases constitutes by far the most critical resistance issue in *Enterobacteriaceae*. Inhibitor-susceptible TEM and SHV penicillinases emerged first in the 1960s, and spread rapidly afterwards. Then, mutations in the catalytic site enabled several TEM and SHV variants to resist to clavulanate or tazobactam, without hydrolyzing cephalosporins (Fig. 1) [33]. Besides, other mutations extended their hydrolysis spectrum to 3GC: these ESBL variants of TEM and SHV were described in the 1980s, soon after the introduction of 3GC [34]. They spread successfully in healthcare-associated strains of *K. pneumoniae*, *Enterobacter* sp. and, in a lesser extent, *E. coli*, causing major hospital outbreaks in the 1990s [35–37]. Nowadays, TEM-type and SHV-type ESBL are still endemic in many hospitals around the world; nevertheless, they tend to be outnumbered by another ESBL class, referred as CTX-M and first described in the early 1990s [38–40]. A key epidemiological aspect of CTX-M-type ESBL is to be mostly found in *E. coli* colonizing subjects with no medical condition, antibiotic exposure, or previous contact with the healthcare setting [41, 42]. This community reservoir fuels a continuous influx of ESBL into the hospital system [43, 44]. The diffusion of CTX-M-producing *Enterobacteriaceae* has been particularly massive in Southeast Asia and Eastern Mediterranean countries (estimated rates of intestinal carriage, ~60% and ~30%, respectively), and traveling in these areas is a major risk factor for carriage acquisition [42, 45, 46]. Carriage rates in the community are now above 5–10% in many other geographic areas [42], and concerns may logically be raised by reports of ESBL-producing *Enterobacteriaceae* (ESBL-PE) in community-acquired UTI [47], intra-abdominal sepsis [13], or even pneumonia [14]. In Europe, at present, 5–15% of critically ill patients are colonized with ESBL-PE at ICU admission [12, 30, 48]; however, very few among them are admitted for a community-acquired ESBL-PE infection [12].

ESBL-PE are resistant to most beta-lactams except cefoxitin, carbapenems and, for a subset of strains, temocillin [49]. Cefoxitin and temocillin have proven efficacy in murine models of urinary sepsis [50, 51], yet clinical data are still pending [52]. Meanwhile, co-resistances to fluoroquinolones, cotrimoxazole and aminoglycosides are commonly observed in ESBL-PE [7], leaving few alternatives to carbapenems for the treatment of severe infections. However, TEM, SHV and CTX-M are all class A beta-lactamases, and many ESBL variants remain susceptible in vitro to beta-lactamase inhibitors. Consequently, the careful use of certain beta-lactam/beta-lactamase inhibitor (BLBLI) associations (namely, amoxicillin–clavulanate, ticarcillin–clavulanate and piperacillin–tazobactam) to treat ESBL-PE with minimal inhibitory concentrations (MIC) ≤8 mg/L is now approved by the European Committee for Antimicrobial Susceptibility Testing (EUCAST), in an attempt to reduce carbapenem consumption and slow down the spread of carbapenem-resistant GNB [53]. Amoxicillin–clavulanate is most frequently inactive (many ESBL-PE coproduce clavulanate-resistant beta-lactamases such as AmpC or OXA-1), and clinical data are lacking for the ticarcillin–clavulanate association, thereby restricting the issue to the use of piperacillin–tazobactam for ESBL-PE with MIC below the aforementioned breakpoint. A meta-analysis published in 2012 reported no statistically significant difference in mortality between carbapenems and BLBLIs administered as either empirical or definite therapy; nevertheless, as underlined by the authors, included studies were heterogeneous and non-randomized, and most of severe patients were initially treated with carbapenems [54]. Piperacillin–tazobactam is probably a suitable carbapenem-sparing option in bacteraemic UTI [55], and shall be safely used in non-urinary sepsis when MICs are ≤2 mg/L [55, 56]. Conversely, an increase in 14-day mortality has been recently reported in patients with ESBL-PE bacteremia (MIC of piperacillin–tazobactam ranging from 4 to 16 mg/L, i.e., the US Clinical Laboratory Standards Institute’s breakpoint) and treated with piperacillin–tazobactam versus carbapenems [57]. The marked inoculum effect observed with tazobactam in ESBL-PE may contribute to explain why in vitro susceptibility does not systematically translate to clinical efficacy [58]. As a whole, the efficacy of BLBLI associations remains scarcely described in severe ESBL-PE infections [59], and has not been specifically investigated in ICU patients (Table 2). In this population, the pharmacokinetic properties of beta-lactams are dramatically modified [60–62], a pivotal point that may lead to sub-optimal BLBLI dosing even for ESBL-PE strains with relatively low MICs. Likewise, and according to the EUCAST guidelines [53], the use of cefepime might be discussed for infections due to ESBL-PE with MIC ≤1 mg/L [63, 64], but high-dose regimen should be used to overstep the risk of sub-optimal concentrations (Table 3) [18].

**Table 2:** Mechanisms of resistance in *Enterobacteriaceae* and non-fermenting Gram-negative bacilli: 10 key-points for the management of antimicrobial therapy in the intensive care unit

1.	Carboxy- and ureido-penicillins should be preferred to 3GC to treat wild-type inducible AmpC-producing *Enterobacteriaceae* (notably *Enterobacter* sp.)
2.	The use of cefepime could be considered as a carbapenem-sparing option in infections due AmpC-hyperproducing *Enterobacteriaceae*
3.	Carbapenems are the first-line choice for severe ESBL-PE infections
4.	The efficacy of BLBLI associations has not been adequately investigated in critically ill patients with ESBL-PE infections: piperacillin–tazobactam might be discussed as a carbapenem-sparing regimen for strains with low MICs (≤2 mg/L), using optimized administration (high doses, extended or continuous infusion, therapeutic drug monitoring) and provided that the source of infection is controlled
5.	In *Pseudomonas aeruginosa*, the rate of resistance emergence under therapy is notably high with imipenem, which should be used only when other beta-lactams are inactive
6.	The empirical use of colistin may be considered in ICU with high prevalence of carbapenemase-producing GNB
7.	Colistin resistance may emerge in carbapenem-resistant GNB after exposure to this drug
8.	Whether combination therapy prevents the emergence of resistance in non-fermenting GNB is not proven
9.	In spite of a strong rational, the ecological benefit of de-escalation remains to be confirmed in adequate prospective studies
10.	The long-term ecological impact of SOD/SDD must be assessed in ICUs with high prevalence of multidrug-resistant GNB

*3GC* third-generation cephalosporins, *ESBL-PE* extended-spectrum beta-lactamase-producing *Enterobacteriaceae*, *BLBLI* beta-lactam/beta-lactamase inhibitor, *MIC* minimal inhibitory concentration, *ICU* intensive care unit, *GNB* Gram-negative bacilli, *SOD/SDD* selective oral decontamination/selective digestive decontamination.

**Table 3:** Antimicrobial agents for the treatment of *Enterobacteriaceae*, *Pseudomonas aeruginosa* and *Acinetobacter baumannii* infections in critically ill patients: MIC breakpoints (European Committee of Antimicrobial Susceptibility Testing, guidelines 2015) and first-line daily doses

Antimicrobial agent	MIC breakpoint (mg/L) for susceptibility			Usual daily dose^a^ (intra-venous)	Comment
	*Enterobacteriaceae*	*P. aeruginosa*	*A. baumannii*		
Piperacillin	≤8	≤16	ND	4 g/6 h	Consider extended or continuous infusion after a LD
Piperacillin–tazobactam	≤8	≤16	ND	4 g–500 mg/6 h	Consider extended or continuous infusion after a LD
Aztreonam	≤1	≤1	IR	2 g/6–8 h	Consider extended or continuous infusion after a LD
Ceftazidime	≤1	≤8	ND	2 g/6–8 h	Consider extended or continuous infusion after a LD
Cefepime	≤1	≤8	ND	1–2 g/8 h	Consider extended or continuous infusion after a LD High doses for *P. aeruginosa* infections
Ertapenem	≤0.5	IR	IR	2 gr/24 h	Once-daily administration
Meropenem	≤2	≤2	≤ 2	1–2 g/8 h	Consider extended infusion after a LD
Imipenem	≤2	≤4	≤ 2	1 g/6–8 h	No extended infusion (instability)
Gentamicin	≤2	≤4	≤ 4	6–8 mg/kg/24 h	Once-daily administration
Tobramycin	≤2	≤4	≤ 4	6–8 mg/kg/24 h	Once-daily administration
Amikacin	≤8	≤8	≤ 8	25–30 mg/kg/24 h	Once-daily administration
Ciprofloxacin	≤0.5	≤0.5	≤ 1	400 mg/8 h	
Colistin	≤2	≤4	≤ 2	4.5 MU/12 h after a LD of 9 MU	Nebulized administration may be considered for VAP
Tigecycline	≤1	IR	ND	50 mg/12 h after a LD of 100 mg	High-dosing regimen (100 mg/12 h after a LD of 200 mg) has been proposed for severe and/or *A. baumannii* infections, notably VAP
Fosfomycin	≤32	ND	ND	ND	High doses may be considered (in combination) for extensively drug-resistant Gram-negative bacilli

Based on references [53], [18], [116], [170], [171] and [172].

Extended infusion means administration over a 3- to 4-h period.

*MIC* minimal inhibitory concentration, *ND* not defined, *IR* intrinsic resistance, *LD* loading dose, *VAP* ventilator-associated pneumonia.

^a^Daily doses of beta-lactams, fluoroquinolones and colistin must be adjusted in patients with renal failure.

In parallel to ESBL, plasmid-borne cephalosporinases have gained increasing prominence in *Enterobacteriaceae*, including in community-acquired strains [21]. These beta-lactamases are actually encoded by chromosomal *bla*_AmpC_ genes of *Enterobacteriaceae* that have been captured on MGE. CMY-2 from *Citrobacter freundii* is the most frequently encountered type [65]. Most of plasmid-borne cephalosporinases confer a similar pattern of resistance to that of derepressed AmpC.

As the prevalence of ESBL and plasmid-borne cephalosporinases rose, so did the consumption of carbapenems, which promoted the emergence of CRE through the diffusion of plasmid-borne carbapenemases. Unfortunately, their story shall be similar to that of ESBL-PE, namely a first step in *K.pneumoniae* (more rarely in *Enterobacter* sp.) affecting hospital settings with local outbreaks (e.g., VIM and KPC) [66, 67], and then emerging in the community in *E. coli* (NDM and OXA-48 variants) [68, 69] (Fig. 1). This new wave after the CTX-M pandemic raises high concerns as CRE are a step ahead of ESBL-PE in terms of multidrug resistance: for most, only colistin, tigecycline and gentamicin (for some KPC-producing strains) still have an activity (Table 3). OXA-48 is an exception as it hydrolyzes penicillins (with or without inhibitor) and carbapenems (low level of resistance), but not 3GC [70]. Yet, many OXA-48-producing *Enterobacteriaceae* coproduce an ESBL, jeopardizing all regular beta-lactam antibiotics [69]. While originally restricted to certain geographic areas (USA and Israel for KPC, Greece and Italy for VIM, India and Pakistan for NDM and the Eastern and Southern Mediterranean area for OXA-48) [71], CRE are currently spreading worldwide through travelers and repatriated patients [72–74], and are now isolated in subjects with no previous stay in endemic areas [75, 76]. Lastly, it should be underlined that carbapenemase production is not the sole mechanism of carbapenem resistance in *Enterobacteriaceae*, since this phenotype may also emerge under therapy in ESBL-PE or AmpC hyperproducers with acquired impermeability to carbapenems due to mutation-derived loss of outer membrane porins [77–79].

#### Resistance to other antimicrobials

Aminoglycosides resistance in *Enterobacteriaceae* mainly relies on AMEs that hamper antibiotic activity by engrafting various radicals (aminoglycoside phosphotransferase, APH, aminoglycoside nucleotidyltransferase, ANT and aminoglycoside acetyltransferase, AAC, see Additional file 1: Table S2). An intrinsic AME production is met in *Providencia stuartii* (AAC(2′), resistance to gentamicin and tobramycin) and *Serratia marcescens* (AAC(6′)-I, low-level resistance to tobramycin and amikacin). Other species are intrinsically susceptible but can acquire AME-encoding genes on plasmids that often carry multiple resistance determinants, including ESBL [80]. Current rates of co-resistance in hospital-acquired ESBL-PE are 50–60% for gentamicin and 10–20% to amikacin [81, 82], although local variations are observed. Methylases of the 16S ribosomal subunit (i.e., the target of aminoglycosides) have been more recently described, notably in NDM-producing strains [83]: these enzymes, named ArmA and Rmt, confer resistance to all aminoglycosides except neomycin.

All *Enterobacteriaceae* are naturally susceptible to quinolones and fluoroquinolones. High-level resistance emerges after successive chromosomal mutations in the DNA gyrase- and topoisomerase IV-encoding genes (*gyrA* and *parC*, respectively), each mutation causing a rise in the MICs [84]. Thus, strains with a single mutation can appear susceptible to fluoroquinolones but highly resistant to quinolones [53]. This phenotype may ease the emergence of mutants with high-level fluoroquinolone resistance under fluoroquinolone monotherapy, especially when the bacterial *inoculum* is high [85]. Chromosomal mutations may also lead to decreased permeability or overexpression of efflux pumps, resulting in reduced susceptibility. Besides mutations, plasmid-encoded resistance has emerged in the 2000s with Qnr (A, B, C, D and S subtypes), a small DNA-mimicking protein that confers low-level fluoroquinolone resistance [86], AAC(6′)-Ib-cr, an AME for which two mutations extend the resistance spectrum to ciprofloxacin and norfloxacin [87], and the QepA efflux pump [88]. It is noteworthy that these plasmid-borne determinants of fluoroquinolone resistance are frequently associated with ESBL [89].

Resistance to colistin, the last-resort antibiotic for CRE infections, is now under scrutiny. *Proteus* sp., *Providencia* sp., *Serratia* sp. and *Morganella* sp. are intrinsically resistant to colistin, and the acquisition of carbapenemase-encoding genes by these species is of major concern [71]. To date, no transferable resistance determinant has been described, and colistin resistance mainly rests on mutations in genes involved in the outer membrane polarity [90]. The spread of colistin-resistant *Enterobacteriaceae*, most notably *K. pneumoniae*, is alarming in environments with high prevalence of CRE, that is, in ICU with high volume of colistin consumption [91].

### Antimicrobial resistance in non-fermenting GNB

#### *Pseudomonas aeruginosa*

Similarly to AmpC-producing *Enterobacteriaceae*, *P. aeruginosa* harbors an inducible AmpC-type cephalosporinase that can be derepressed following mutations in the regulation system [92]. Wild-type strains of *P. aeruginosa* are resistant to amoxicillin (with or without clavulanate), 1GC, 2GC, cefotaxime, ceftriaxone and ertapenem, while they remain susceptible to ticarcillin, piperacillin, ceftazidime, cefepime, imipenem, meropenem and doripenem. Aztreonam activity is variable. Unlike tazobactam, clavulanate is a strong inducer of AmpC in *P. aeruginosa*, and experimental data suggest a risk of clinical failure with the ticarcillin–clavulanate association [93]. AmpC-hyperproducing strains remain susceptible to carbapenems only.

*P. aeruginosa* has several three-component efflux systems, some of which confer resistance to beta-lactams when strongly expressed after mutations in their promoter regions (Table 4) [94]. The most frequently involved system is MexAB-OprM, whose overexpression confers resistance to ticarcillin, aztreonam, cefepime and meropenem. Efflux pumps are major determinants of the multidrug resistance phenotypes that are increasingly observed in *P. aeruginosa*. A key feature is that different antimicrobial classes may be substrates of a single pump: exposure to a given class (e.g., beta-lactams) may thereby select mutants with resistance to other classes (e.g., beta-lactams plus fluoroquinolones or aminoglycosides) [95].

**Table 4:** Main mechanisms of acquired antimicrobial resistance in *Pseudomonas aeruginosa*

Mechanism	Genetic event	Antimicrobials
High-level expressed AmpC cephalosporinase	Chromosomal mutation	Penicillins (with or without beta-lactamase inhibitors), cephalosporins, aztreonam
Other beta-lactamases		
Penicillinases^a^	MGE acquisition	Penicillins
Extended-spectrum beta-lactamases^b^		Penicillins, cephalosporins, aztreonam
Metallo-beta-lactamases^c^ (carbapenemases)		Penicillins, cephalosporins, carbapenems
Loss of OprD (impermeability)	Chromosomal mutation	Imipenem
Active efflux pumps		
MexAB-OprM	Chromosomal mutation	Ticarcillin, cephalosporins, aztreonam, meropenem, fluoroquinolones
MexXY-OprM		Cefepime (±penicillins), aminoglycosides, fluoroquinolones
MexEF-OprN		Meropenem, fluoroquinolones
MexCD-OprJ		Cefepime, aztreonam (+/− penicillins), fluoroquinolones
Aminoglycoside-modifying enzymes^d^	MGE acquisition	Aminoglycosides
16S rRNA methylases	MGE acquisition	Aminoglycosides
Topoisomerases modifications	Chromosomal mutation	Fluoroquinolones
Lipid A (LPS) modifications	Chromosomal mutation	Polymyxins

*MGE* mobile genetic element (plasmid or transposon).

Most common enzyme types: ^a^PSE and OXA; ^b^PER, SHV, GES and OXA; ^c^VIM and IMP (SIM, GIM and SPM types are less common); ^d^AAC(3)-I, AAC(3)-II, AAC(6′)-I, AAC(6′)-II and ANT(2′)-I.

Imipenem resistance in otherwise beta-lactam-susceptible strains of *P. aeruginosa* indicates the functional loss of OprD, a porin which manages the passage of imipenem through the outer membrane [95, 96]. The emergence of imipenem resistance under therapy results almost exclusively from the selection of OprD mutants, either from a previously imipenem-susceptible *inoculum* or, more occasionally, after cross-transmission of another clone [97]. The risk appears notably high in clinical practice. Indeed, in four randomized controlled trials (RCTs) including patients with hospital-acquired *P. aeruginosa* pneumonia, the average rate of resistance emergence under therapy was 30% (range, 6–53%) for imipenem, while only 15% (range, 6–36%) for other beta-lactams [98].

*P. aeruginosa* has the ability to develop resistance to all beta-lactams as the sole result of chromosomal mutations. Nonetheless, the species can acquire MGE-encoded beta-lactamases, including ESBL and carbapenemases (Table 4) [96]. Major hospital outbreaks have notably been observed with VIM or IMP carbapenemase-producing clones [99, 100].

Resistance to tobramycin mostly occurs through the acquisition of AMEs, while resistance to amikacin mostly depends on the over-expression of efflux pumps [101]. MGE-borne 16S rRNA methylases such as ArmA, RmtA and RmtD are also reported as an emerging mechanism of aminoglycoside resistance in *P. aeruginosa* [102]. Fluoroquinolone resistance results from mutations in the topoisomerase-encoding genes and/or the hyper-expression of efflux systems [95]. Lastly, and as for *Enterobacteriaceae*, colistin-resistant mutants of *P. aeruginosa* may emerge in settings with high frequency of colistin use [90].

#### *Acinetobacter baumannii*

*Acinetobacter baumannii* naturally produces a non-inducible AmpC-type cephalosporinase (ACE-1 or ACE-2) and an OXA-51-like oxacillinase which confer, at basal levels of expression, intrinsic resistance to aminopenicillins, 1GC, 2GC and aztreonam [103]. Ertapenem naturally lacks activity against *A. baumannii*. Together with a marked impermeability and the expression of multiple efflux systems, the plasticity of its genome enables the species to gather many resistance mechanisms, leading easily to multidrug resistance (Table 5). Most of the time, acquired resistance to carboxypenicillins, ureidopenicillins and 3GC rests on the overproduction of the AmpC-type cephalosporinase. However, in addition to plasmidic narrow-spectrum beta-lactamases, several ESBLs have also been acquired by *A. baumannii*: PER and VEB are the most frequently encountered types, particularly within pandemic clones [104]. In both cases, imipenem and meropenem remain the drugs of choice. More worrying are the emergence and dissemination of carbapenem-resistant clones since the end of the 1980s. Although carbapenem resistance can result from the over-expression of the chromosomal OXA-51-like enzyme [105], this phenotype is mostly due to the acquisition of plasmid-borne OXA-23-like, IMP, VIM, SIM or, more recently, NDM-type carbapenemases [102]. Of note, the prevalence of such carbapenemase-producing strains increases steadily from Northern to Southern European countries [104]. Acquired resistances to fluoroquinolones (mutations in *gyrA* and/or *parC*) and aminoglycosides (plasmid-borne AMEs—particularly AAC(3), AAC(6′) and APH(3′)—and 16S rRNA methylases) are commonly observed in ESBL- as well as carbapenemase-producing *A. baumannii* strains.

**Table 5:** Main mechanisms of acquired antimicrobial resistance in *Acinetobacter baumannii*

Mechanism	Genetic event	Antimicrobials
High-level expressed AmpC cephalosporinase	Chromosomal mutation	Penicillins (with or without beta-lactamase inhibitors), 3GC
High-level expressed OXA-51-like beta-lactamase	Chromosomal mutation (insertion of IS*Aba1* upstream of *bla*_*OXA*-*51*_)	Carbapenems
Other beta-lactamases		
Extended-spectrum beta-lactamases^a^	MGE acquisition	Penicillins, 3GC
Metallo-beta-lactamases^b^ (carbapenemases)		Penicillins, 3GC, carbapenems
Oxacillinase-type carbapenemases^3^		Penicillins, carbapenems
Functional loss of porins (impermeability)	Chromosomal mutation	Variable
Altered penicillin-binding proteins	Chromosomal mutation	Variable
Active efflux pumps		
AdeABC	Chromosomal mutation	Beta-lactams (variable), aminoglycosides, fluoroquinolones, tigecycline
AdeM		Aminoglycosides, fluoroquinolones
AdeIJK		Tigecycline
Aminoglycoside-modifying enzymes^d^	MGE acquisition	Aminoglycosides
16S rRNA methylases	MGE acquisition	Aminoglycosides
Topoisomerases modifications	Chromosomal mutation	Fluoroquinolones
Lipid A (LPS) modifications	Chromosomal mutation	Polymyxins

*MGE* mobile genetic element (plasmid or transposon), *3GC* third-generation cephalosporins.

Most common enzyme types: ^a^PER, VEB and GES (TEM, SHV and CTX-M are rare in *A. baumannii*); ^b^VIM, SIM, IMP and NDM; ^c^OXA-23-, OXA-40-, OXA-58-, OXA-143 and OXA-235-like; ^d^AAC(3), AAC(6′) and APH(3′).

Colistin stands as the main therapeutic option for ICU-acquired infections due to extensively drug-resistant *A. baumannii*, and should be considered as part of the empirical antibiotic regimen in settings with high densities of carbapenem-resistant strains [106]. Nevertheless, colistin-resistant isolates are now increasingly reported worldwide, especially in patients previously exposed to this drug [107]. This phenotype mainly depends on the loss of lipopolysaccharide (LPS) production secondary to the insertion of the ISA*ba*11 sequence in genes encoding the lipid A biosynthesis [108]. Increased expression of the PmrAB two-component regulatory system is another mechanism of LPS alteration resulting in colistin resistance [109]. Interestingly, the reduction of the negative charge of the lipid A, which lowers the affinity for colistin (positively charged), may also induce cross-resistance to host cationic antimicrobials such as lysozyme [110]. Furthermore, colistin exposure may select for a resistant fraction among an otherwise colistin-susceptible *A. baumannii* population [111, 112]. The prevalence of this mechanism of resistance—referred as heteroresistance—is poorly documented due to missed detection by conventional microbiological methods but could have significant clinical consequences [113].

For infection due to colistin-susceptible *A. baumannii* strains, the benefit of combination with rifampin has not been confirmed by a recent RCT [114]. Sulbactam, a BLI with intrinsic activity against *A. baumannii*, may be useful alone or in combination [103], although clinical data are still scarce. Clinical experience is also limited for minocycline, despite of a high in vitro activity against multidrug-resistant isolates [115]. The use of tigecycline may be discussed in the absence of other option (i.e., colistin resistance or toxicity) [104]: double-dose regimens appear well tolerated and could be more active than standard dosing owing to pharmacokinetic considerations, notably in patients with VAP [116].

#### *Stenotrophomonas maltophilia*

*S. maltophilia* is an emerging pathogen responsible for hospital-acquired infections in patients previously exposed to carbapenems or other broad-spectrum antibiotics [117, 118]. Its intrinsic multidrug resistance phenotype involves several chromosomal determinants. First, the species expresses various efflux systems and most notably the SmeDEF pump, which takes part in the extrusion of certain beta-lactams, quinolones and aminoglycosides [118]. Also, this GNB should be considered as naturally resistant to aminoglycosides, owing to the presence of a chromosomal AAC(6′)-Iz and the thermo-dependent permeability of its outer membrane to this antimicrobial class [119, 120]. Next, *S. maltophilia* produces two chromosomal beta-lactamases, namely, the inducible L1 carbapenemase (conferring an intrinsic resistance to all carbapenems) and the inducible, inhibitor-susceptible L2 cephalosporinase. Together, these enzymes may confer various resistance phenotypes, according to their respective degrees of expression and the concomitant levels of impermeability and efflux [118, 121]. The ticarcillin–clavulanate association remains usually the most effective beta-lactam regimen, while cephalosporins are almost constantly inactive.

*S. maltophilia* is highly susceptible to the trimethoprim–sulfamethoxazole combination, which is traditionally seen as the cornerstone of therapy [121]. Acquired resistance is however reported with various frequencies and rests on dihydropteroate synthases encoded by the MGE-borne *sul* genes. Fluoroquinolones, particularly ciprofloxacin, levofloxacin and moxifloxacin, are active despite the low-level expression of a Qnr protein encoded by the chromosomal *SmQnr* gene [122]. High-level resistance to fluoroquinolones may emerge through the selection of mutants with increased expression of SmQnr proteins or efflux pumps (SmeDEF or SmeVWX) [123].

The association of trimethoprim–sulfamethoxazole (high-dosing regimen) with ticarcillin–clavulanate or fluoroquinolones is generally advocated as a first-line regimen for serious infections [121]. Indeed, synergy with these combinations is observed in vitro for more than half of isolates [124]. Alternatives include monotherapy with trimethoprim–sulfamethoxazole, fluoroquinolones, or tigecycline [125, 126], with a possible synergic effect when the latter is associated to colistin [127, 128].

### Is administration of combination therapy needed to prevent resistance?

To increase the likelihood of adequate coverage, the empirical antimicrobial regimen for VAP or other ICU-acquired infections in patients at risk for multidrug-resistant GNB usually combines a broad-spectrum beta-lactam with anti-pseudomonal activity and either an aminoglycoside or an anti-pseudomonal fluoroquinolone [29]. However, when both agents are active, the benefit of combination therapy over adequate monotherapy has not been proven in terms of clinical cure or microbiological eradication [129–131]. Convincing evidence is similarly lacking to support the routine use of antimicrobial combinations (including a beta-lactam) as definite regimen in an attempt to prevent the emergence of resistance under therapy [129, 132–134]. In *P. aeruginosa* infections, adding an aminoglycoside to an effective beta-lactam does not prevent from the emergence of beta-lactam resistance [133, 135, 136], including in patients treated with imipenem [137]. In *Enterobacteriaceae*, the main mechanism of acquired beta-lactam resistance under therapy is chromosomal AmpC derepression. In a prospective cohort of 218 patients infected with natural AmpC producers and receiving 3GC, the emergence of 3GC resistance was observed in 11 cases (5%): combining 3GC with an aminoglycoside or a fluoroquinolone did not significantly reduce the rate of mutant selection [138]. Therefore, once susceptibility testing results are known, monotherapy with the most active beta-lactam could be considered, with high-dosing regimen and optimized administration (Table 3). Clinical data remain scarce for infections due to multidrug-resistant *A. baumannii* [139], although in vitro studies indicate that combining colistin with rifampicine, carbapenems or tigecycline may be effective to prevent the emergence of colistin-resistant mutants [109].

### A pivotal role for the gut microbiota

The intestinal microbiota forms the main reservoir of multidrug-resistant GNB in critically ill patients [30, 31, 140]. While similar data are currently not available for ICU-acquired infections, some studies have showed that high intestinal densities of resistant bacteria increase the risk of intestinal translocation [141], urinary tract infections [142] and cross-transmission [143]. Antibiotics that reach this microbiome promote the growth of resistant bacteria over the susceptible ones, and each daily dose may exert a significant impact in terms of selective pressure [144]. This appears notably relevant for carbapenems [26], fluoroquinolones [145] or cephalosporins with biliary excretion such as ceftriaxone [146]. Extended treatment with colistin has also been shown to increase the likelihood of colonization with colistin-resistant GNB, including both mutants from otherwise colistin-susceptible species, and intrinsically colistin-resistant *Enterobacteriaceae* [147].

The spectrum, duration of exposure and fecal concentration of the antibiotic may all play a role. Therefore, and although the ecological benefit of such an approach remains to be formally demonstrated [148], de-escalation to the antimicrobial regimen with the narrower spectrum and the lower intestinal excretion should be logically discussed when culture and susceptibility testing results become available. In this respect, new phenotypic and molecular diagnostic tools may fasten the detection of multidrug-resistant GNB—or rule them out precociously—thereby assisting ICU physicians for earlier adjustments of broad-spectrum empirical regimen [149–151].

Another unresolved issue is whether selective oral or digestive decontamination (SOD/SDD) with colistin and/or aminoglycosides compromises the efficacy of these agents by selecting resistant GNB in the ICU. SOD and SDD are infection prevention measures with proven efficacy in reducing the incidence of ICU-acquired bacteremia [152], the all-cause mortality rate at day 28 [153] and, for SDD combined with systemic antibiotic administration, the mortality attributable to VAP [154]. In a meta-analysis published in 2013, SOD and SDD were not associated with an increased risk of acquisition of aminoglycoside-resistant GNB, and were even protective against the acquisition of polymyxin-resistant GNB when compared to standard care [155]. However, the number of included studies was relatively low, most of them were conducted in the 1990s—that is, one decade before the pandemic of carbapenemase-producing *Enterobacteriaceae*—and carriage samples were pooled with clinical samples to assess the acquisition rates of resistant GNB, making the authors conclude that the impact of SOD/SDD on ICU-level antimicrobial resistance rates was understudied. In a recent RCT conducted in Dutch ICUs with low levels of resistance, the use of a tobramycin-based SDD regimen was associated with a gradual increase in the prevalence of aminoglycoside-resistant GNB when compared to SOD [156]. More strikingly, a worrisome rise in the rates of colistin and aminoglycoside resistance has been observed following the implementation of SOD/SDD policies with these antibiotics in ICUs facing outbreaks of ESBL- or carbapenemase-producing *K. pneumoniae* [157–159]. Conversely, two prospective trials reported that colistin-based SDD regimen might help eradicating CRE carriage [160, 161]. Overall, pending further longitudinal studies, SOD/SDD should probably be used with caution in environments with high prevalence of multidrug-resistant GNB to preserve the efficacy of polymyxins and aminoglycosides as last-resort agents [162, 163].

## Concluding remarks and perspectives

The spread of multidrug-resistant GNB in the hospital setting is now seen as a globalized threat [15], and ICU patients are especially exposed to the risk. The number of potential novel agents in the pipeline is low; nevertheless, the development of new BLBLI combinations may raise significant hopes [164]. Avibactam (NXL104) is a synthetic BLI with activity on Ambler’s class A (including ESBL and KPC-type carbapenemases), class C (derepressed chromosomal AmpC or plasmid-borne AmpC) and some class D (oxacillinases) beta-lactamases [165]. In vitro, ceftazidime–avibactam, ceftaroline–avibactam and aztreonam–avibactam associations have shown promising results against 3GC-resistant and even KPC-producing *Enterobacteriaceae* [166, 167]. Avibactam also lessens the MICs of ceftazidime in AmpC-hyperproducing *P. aeruginosa* [168]. Other cephalosporin-BLI associations such as cefepime–tazobactam, ceftriaxone–sulbactam and ceftolozane–tazobactam are under evaluation [59, 169]. Clinical works addressing the yield of these new combinations in the ICU are highly warranted. Besides, improvements in the use of already available drugs are still possible (e.g., piperacillin–tazobactam for ESBL-PE with low MICs, or colistin for carbapenem-resistant GNB), both when indications and modalities of administration (including therapeutic drug monitoring) are considered. Unfortunately, the emergence of bacterial resistance following the introduction of new drugs appears as an unavoidable and endless process and every initiative aiming at limiting the selective pressure of antibiotics on the intestinal flora is more than ever justified.

## Additional file

Additional file 1:
**Table S1.** Intrinsic beta-lactam resistance in clinically relevant *Enterobacteriaceae* species. **Table S2.** Aminoglycoside-modifying enzymes in Gram-negative bacilli: main clinically relevant types and corresponding resistance profiles.

## Abbreviations

AAC: aminoglycoside acetyltransferase
AME: aminoglycoside-modifying enzyme
ANT: aminoglycoside nucleotidyltransferase
APH: aminoglycoside phosphotransferase
CRE: carbapenem-resistant *Enterobacteriaceae*
BLBLI: beta-lactam/beta-lactamase inhibitor
ESBL: extended-spectrum beta-lactamase
ESBL-PE: extended-spectrum beta-lactamase-producing *Enterobacteriaceae*
1GC: first-generation cephalosporin
2GC: second-generation cephalosporin
3GC: third-generation cephalosporin
GNB: Gram-negative bacilli
ICU: intensive care unit
MGE: mobile genetic element
MIC: minimal inhibitory concentration
LPS: lipopolysaccharide
RCT: randomized controlled trial
RNA: ribonucleic acid
SOD: selective oral decontamination
SDD: selective digestive decontamination
UTI: urinary tract infection
VAP: ventilator-associated pneumonia
