# Supplementary material for: Mechanisms of antimicrobial resistance in Gram-negative bacilli
Source: Ann Intensive Care. 2015 Aug 12;5:21. doi: 10.1186/s13613-015-0061-0 (PMC4531117; doi:10.1186/s13613-015-0061-0)
Supplement: Additional file 1: — Table S1. Intrinsic beta-lactam resistance in clinically relevant Enterobacteriaceae species. Table S2. Aminoglycoside-modifying enzymes in Gram-negative bacilli: main clinically relevant types and corresponding resistance profiles. [file 13613_2015_61_MOESM1_ESM.doc]

**Electronic Supplementary Material**

**MECHANISMS OF Antimicrobial resistance**

**in Gram-negative bacilli**

**Étienne Ruppé, Paul-Louis Woerther, François Barbier**

| **Chromosomal beta-lactamase** | | None | Non-inductible AmpC cephalosporinase (low-level expression) | Inhibitor-susceptible penicillinase | Inductible AmpC cephalosporinase | Non-inductible, inhibitor-susceptible cephalopsorinase |
| --- | --- | --- | --- | --- | --- | --- |
| **Species** | | *Proteus mirabilis*  *Salmonella enterica* | *Escherichia coli*  *Shigella* sp | *Klebsiella pneumoniae*  *Klebsiella oxytoca*  *Citrobacter koseri* | *Enterobacter aerogenes*  *Enterobacter cloacae*  *Serratia marcescens*  *Hafnia alvei*  *Morganella morganii*  *Providencia stuartii*  *Citrobacter freundii* | *Proteus vulgaris*  *Proteus penneri* |
| **Aminopenicillins** | Amoxicillin | S | S | R | R | R |
|  | Amoxicillin – Clavulanic acid | S | S | S | R | S |
| **Carboxypenicillins** | Ticarcillin | S | S | R | S | S |
|  | Ticarcillin – Clavulanic acid | S | S | S | S | S |
| **Ureidopenicillins** | Piperacillin | S | S | DS/R | S | S |
|  | Piperacillin – Tazobactam | S | S | S | S | S |
| **Cephamycin** | Cefoxitin | S | S | S | R | R |
| **1GC** | Cefalotin | S | S | DS/R | R | R |
| **2GC** | Cefamandole | S | S | S | DS/R | S |
| **3GC** | Cefotaxime, Ceftriaxone | S | S | S | S | S |
|  | Ceftazidime | S | S | S | S | S |
| **4GC** | Cefepime | S | S | S | S | S |
| **Monobactam** | Aztreonam | S | S | S | S | S |
| **Carbapenems** | Ertapenem | S | S | S | S | S |
|  | Imipenem | S | S | S | S | S |
|  | Meropenem | S | S | S | S | S |

**Table S1.** Intrinsic beta-lactam resistance in clinically relevant *Enterobacteriaceae* specie

S, susceptible; DS, decreased susceptibility; R, resistant; 1GC, 2GC, 3GC and 4GC means first-, second-, third- and fourth-generation cephalosporins, respectively.

**Table S2.** Aminoglycoside-modifying enzymes in Gram-negative bacilli: main clinically relevant types and corresponding resistance profiles

| **Enzymes** | **Gentamicin** | **Tobramycin** | **Amikacin** |
| --- | --- | --- | --- |
| **APH(3’)** | S | S | R |
| **AAC(2’)** | S/R | S/R | S |
| **AAC(3) - I** | R | S | S |
| **AAC(3) - II/IV** | R | R | S |
| **AAC(6’) - I** | S | R | S/R |
| **AAC(6’) - II** | R | R | S |
| **ANT(2’’)** | R | S/R | S |
| **ANT(4’) - II** | S | S/R | S/R |

S, susceptible; R, resistant; APH, aminoglycoside O-phosphotransferase ; AAC, aminoglycoside N-acetyltransferase ; ANT, aminoglycoside O-nucleotidyltransferase.
